# Minimally Invasive Scoliosis Surgery: A Novel Technique in Patients with Neuromuscular Scoliosis

**DOI:** 10.1155/2015/481945

**Published:** 2015-11-16

**Authors:** Vishal Sarwahi, Terry Amaral, Stephen Wendolowski, Rachel Gecelter, Melanie Gambassi, Christos Plakas, Benita Liao, Sarika Kalantre, Chhavi Katyal

**Affiliations:** ^1^Division of Pediatric Orthopedics, Cohen Children's Medical Center, New Hyde Park, NY 11040, USA; ^2^Department of Orthopaedic Surgery, Children's Hospital at Montefiore and Albert Einstein College of Medicine, Bronx, NY 10467, USA; ^3^Department of Pediatric Anesthesiology, Cohen Children's Medical Center, New Hyde Park, NY 11040, USA; ^4^Division of Pediatric Cardiology, Children's Hospital at Montefiore and Albert Einstein College of Medicine, Bronx, NY 10467, USA; ^5^Division of Critical Care, Department of Pediatrics, Children's Hospital at Montefiore and Albert Einstein College of Medicine, Bronx, NY 10467, USA

## Abstract

Minimally invasive surgery (MIS) has been described in the treatment of adolescent idiopathic scoliosis (AIS) and adult scoliosis. The advantages of this approach include less blood loss, shorter hospital stay, earlier mobilization, less tissue disruption, and relatively less pain. However, despite these significant benefits, MIS approach has not been reported in neuromuscular scoliosis patients. This is possibly due to concerns with longer surgery time, which is further increased due to more levels fused and instrumented, challenges of pelvic fixation, size and number of incisions, and prolonged anesthesia. We modified the MIS approach utilized in our AIS patients to be implemented in our neuromuscular patients. Our technique allows easy passage of contoured rods, placement of pedicle screws without image guidance, partial/complete facet resection, and all standard reduction maneuvers. Operative time needed to complete this surgery is comparable to the standard procedure and the majority of our patients have been extubated at the end of procedure, spending 1 day in the PICU and 5-6 days in the hospital. We feel that MIS is not only a feasible but also a superior option in patients with neuromuscular scoliosis. Long-term results are unavailable; however, short-term results have shown multiple benefits of this approach and fewer limitations.

## 1. Introduction

MIS technique in AIS has been shown to provide similar correction and pedicle screw accuracy with lower blood loss and rate of transfusion [1]. In adult scoliosis patients, short-term benefits such as less pain, narcotics usage, and shorter hospital stay have also been reported [2]. These benefits seem ideal for patients with neuromuscular scoliosis, who routinely spend more days in the ICU and in the hospital, require prolonged intubation, have increased blood loss, and require multiple units of blood transfusion [3–5]. However, curves in children with neuromuscular scoliosis are usually larger and stiffer, with more number of levels fused and instrumented (16–18) and a need for pelvic fixation [5]. The length and type of skin incision are also important. The standard stab incision for placement of percutaneous pedicle screws cannot be utilized as passing a rod that is contoured in the normal sagittal profile (thoracic kyphosis and lumbar lordosis) will be quite challenging and thirty-two to thirty-eight stab incisions in the back for screw insertion are less than desired.

In MIS technique for AIS patients, the skin incisions have been modified to three noncontiguous midline incisions through which 2–4 levels (4–8 screws) can be fused utilizing a muscle splitting approach [6]. The pedicle screws are inserted in a free hand anatomic manner obviating the need for fluoroscopy and the technique allows for partial or complete facet osteotomy/excision [6]. Adequate fusion has been documented on CT scans as the fusion bed comprises the facet joint, pars, and variable areas of transverse process and lamina while preserving the midline osteoligamentous complex [6]. This approach also allows multiple reduction maneuvers, including rod translation, rod derotation, in situ bending, direct vertebral rotation, and spine translation [6]. However, despite all the advantages and feasibility of the technique, MIS approach in AIS takes much longer, which has thus far limited its application in neuromuscular scoliosis.

The purpose of this study is to detail a modification of the previously described technique for minimally invasive posterior spinal fusion, allowing its application in the neuromuscular scoliosis patient population with comparable length of surgery.

## 2. Materials and Methods

We have been utilizing minimally/less invasive spine surgery techniques in neuromuscular scoliosis patients for around one year. This technique allows for all standard reduction maneuvers, rod insertion, free hand anatomic insertion of pedicle screws with/without image guidance, adequate facet osteotomy to enable fusion, facet joint excision for stiffer curves, and pelvic fixation.

### 2.1. Surgical Technique

**Figure 1:**
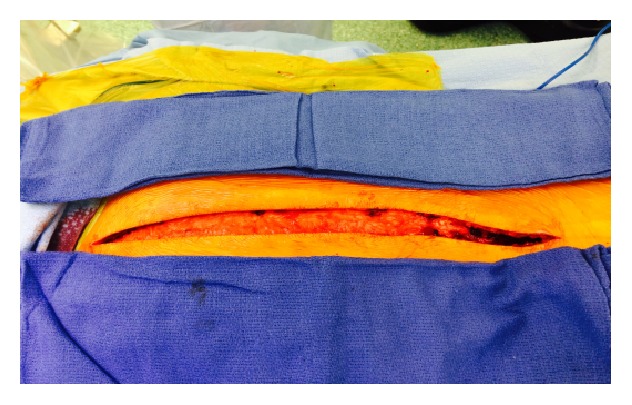
Intraoperative image showing the modification of skin incision to facilitate instrumentation.

A single midline curvilinear skin incision is made for instrumentation of sixteen to eighteen segments following the deformity (Figure 1).

**Figure 2:**
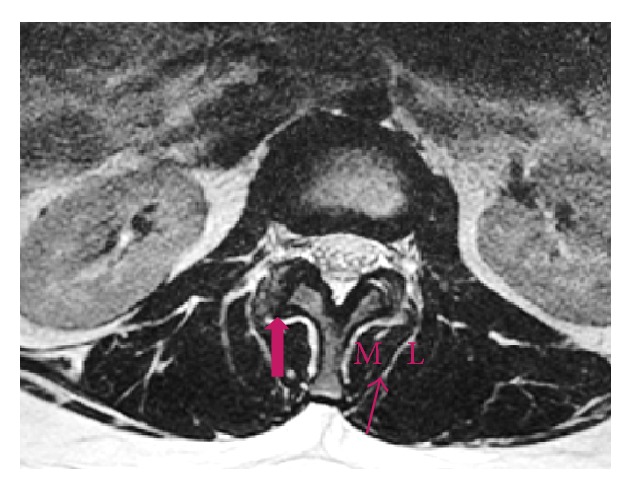
MRI axial image of the lumbar spine with the block arrow pointing to the facet joint, which is the most prominent area after the spinous process and is easily palpable after the lumbar fascia is incised. The thin arrow denotes the plane of dissection between multifidus medially (M) and longissimus laterally (L). This plane is approximately 2 to 2.5 cm lateral to the midline.

**Figure 3:**
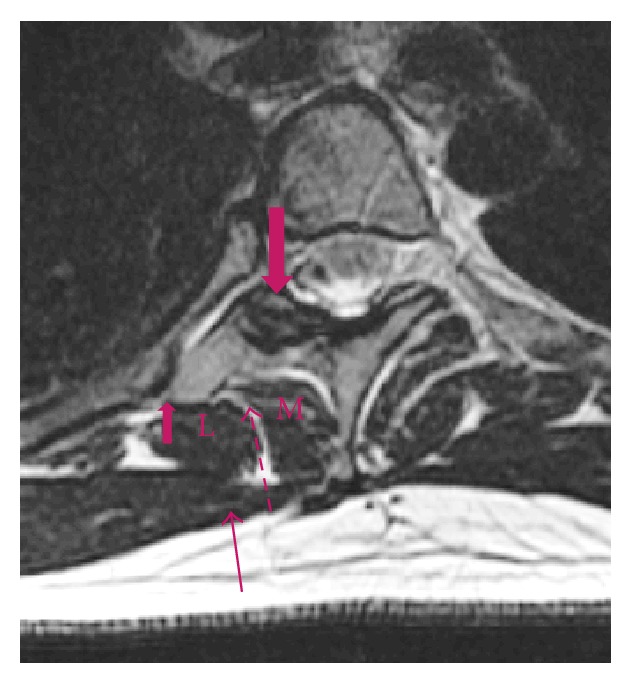
MRI axial image of the thoracic spine showing the facet joint (large block arrow), which is less prominent than the transverse process (small block arrow). The plane of dissection (dashed arrow) is between multifidus medially (M) and longissimus laterally (L). Also notice the fibers running transversely of the trapezius, which lies superficially to the thoracodorsal fascia and has to be incised to expose the surgical plane.

The skin is undermined and mobilized on either side of midline to allow for the placement of pedicle screws on both sides. The muscle dissection is started in the lumbar spine, where the facet can be manually palpated. A stab incision in the fascia is made, directly over the facet, and muscle plane is developed bluntly between multifidus and longissimus coli with a Cobb elevator or an insulated electrocautery (Figures 2 and 3).

The facet joint is exposed and a small Gelpi retractor is inserted. Further dissection is carried out as needed to expose the adjacent transverse process and portion of the lamina. We take advantage of the overlapping spine anatomy in locating the facet joints at the level above and at the level below. The midline osteoligamentous structures are preserved. Further dissection is extended caudally and cranially to expose additional levels. This approach thus creates a paramedian exposure similar to Wiltse approach along the entire length of the spine and also the posterior superior iliac spine. In the thoracic spine, the fibers of latissimus dorsi and trapezius need to be dissected before reaching the thoracodorsal fascia. The longitudinal fibers can be split after the fascia is incised to expose the facet joint. After the facet joint is exposed, a facetectomy is performed with either a 1/4′′ osteotome or a high speed burr. Adequate excision of the facet joint is carried out to ensure a solid fusion. The adjacent transverse process, pars, and portion of lamina are decorticated to create a sizeable fusion bed after the muscle fibers are elevated with Bovie if needed. The fusion bed thus extends from the tip of the transverse process laterally to lamina medially and includes the facet joint and pars interarticularis. This fusion bed area reaches comparable size to standard techniques except for the spinous process, which is often excised by surgeons in the standard technique.

**Figure 4:**
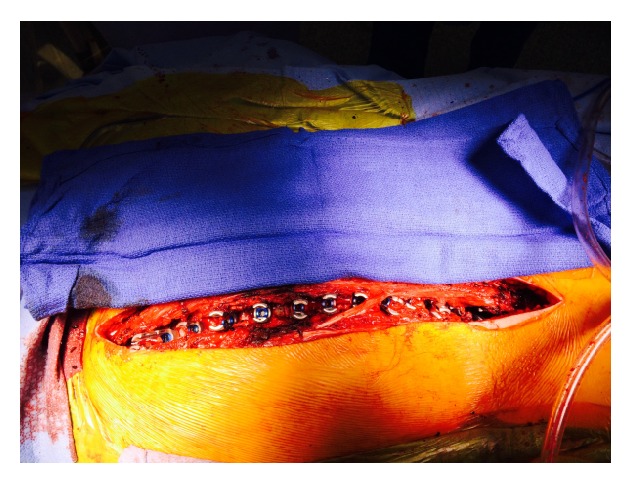
Intraoperative image showing pedicle screws that have been inserted utilizing free hand anatomic technique on the left side at multiple levels.

Pedicle screws are placed utilizing the free hand technique but can be inserted under fluoroscopy (Figure 4).

**Figure 5:**
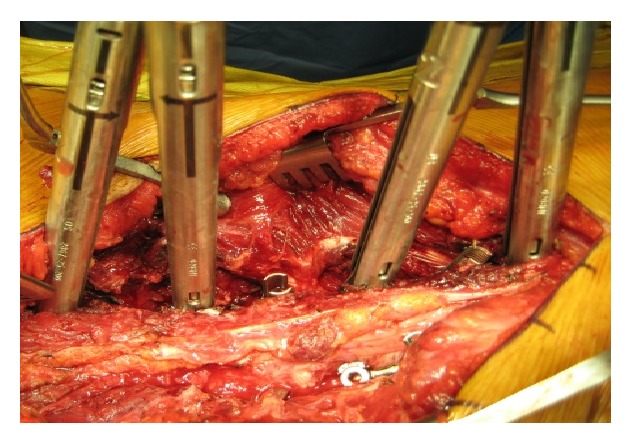
A close-up view of the lumbar spine with rod reduction instruments attached to the pedicle screws at the time of rod insertion.

**Figure 6:**
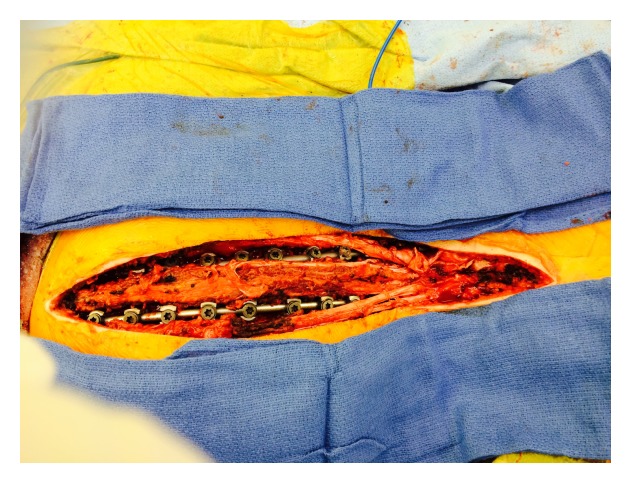
Rods have been inserted on both of the sides and correction has been obtained. Notice that the midline osteoligamentous structures have been preserved.

Complete exposure of the transverse process is usually not needed to identify the entry point but can be carried out if desired. Sacral screw insertion is carried out in a free hand manner but can also be done under fluoroscopy for a tricortical approach. Pelvic fixation can be carried out similarly. The PSIS serves as an attachment for multifidus and thus the dissection plane leads to the entry point. We prefer to place pelvic screws under intermittent fluoroscopy to direct the screw towards dense bone superior to the sciatic notch. Two rods, cut to appropriate length, are contoured in the normal sagittal plane and are inserted caudad to cephalad. We start with the concave rod first. A rod translation or rod derotation maneuver can be carried out to seat the rod compression and distraction as needed can also be carried out. In situ rod contouring can also be carried out but is not our preference. Direct vertebral rotation maneuver is then carried out off the concave-side screws (Figures 5 and 6).

A mix of allograft and autograft mixed with vancomycin powder is layered after burring the posterior portion of spine. Closure is fairly rapid and is carried out in a layered fashion. We utilize subcutaneous medium hemovac drain. Intraoperative anteroposterior and lateral X-rays are taken to confirm adequate correction. An assessment is made at the end of the case about extubating the patient and in most cases is carried out in the operating room. We prefer to use Ketorolac (Toradol, Roche Laboratories, Nutley, NJ) rather than morphine for analgesia. We do not brace our patients and child is allowed sitting and/or wheelchair transfers next day. Activity is increased gradually as tolerated within the limits of pain. Patients can return to school in 3-4 weeks.

## 3. Results

**Figure 7:**
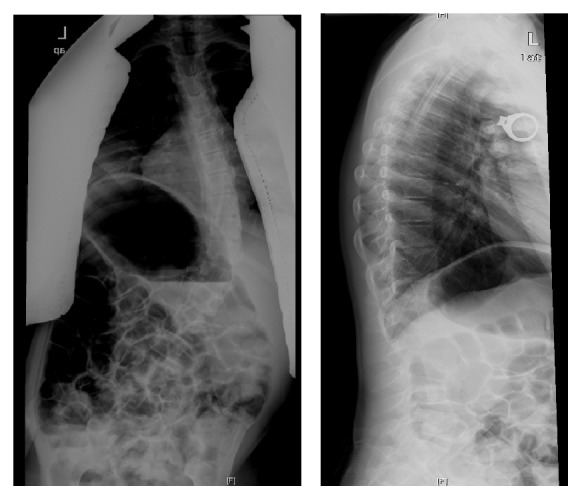
Preoperative radiographs showing a 54-degree scoliosis deformity in a patient with Rett syndrome. Patient has coronal imbalance. The pelvis is level.

We present two case examples to show the degree of correction and benefits of MIS technique in neuromuscular scoliosis. In our first case, the patient is an 11-year-old girl with a 54° right-sided long C shaped curve (Figure 7).

**Figure 8:**
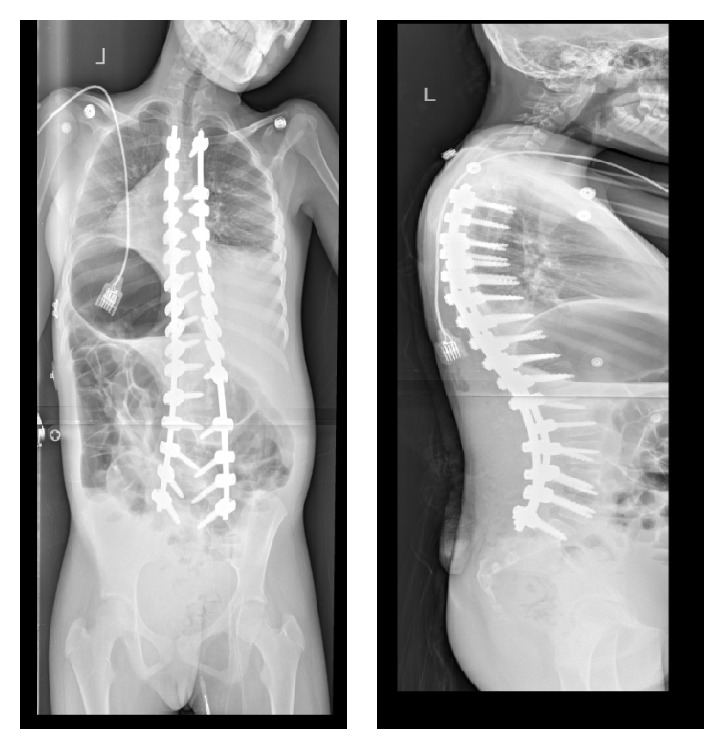
Immediate postoperative radiographic images showing excellent correction of the deformity. Patient is well-balanced in coronal and sagittal planes. The pelvis and shoulder are level.

She had underlying diagnoses of Rett syndrome. Patient was noncommunicative but was able to ambulate independently. Patient had minimal pelvic obliquity, 5.4 cm of coronal imbalance, and normal sagittal parameters. Patient underwent posterior spine fusion from T3-S1 utilizing pedicle screw instrumentation and the approach described above. Multimodality neuromonitoring was utilized with no changes throughout the surgery. Total duration was 5 hours and estimated blood loss was 600 mL. Patient received 1 unit of packed red blood cells intraoperatively and was successfully extubated at the end of the procedure. Her PICU stay was one day and she was able to get out of bed and ambulate with a walker on POD #2. Her pain was controlled with Tylenol and Toradol around the clock, with oxycodone for breakthrough pain. She was discharged home on POD #4 with her pain controlled on oral medication. At most recent follow-up, she was at her preoperative levels of activity, ambulating, and with no significant pain. Postoperative X-rays are shown in Figure 8.

**Figure 9:**
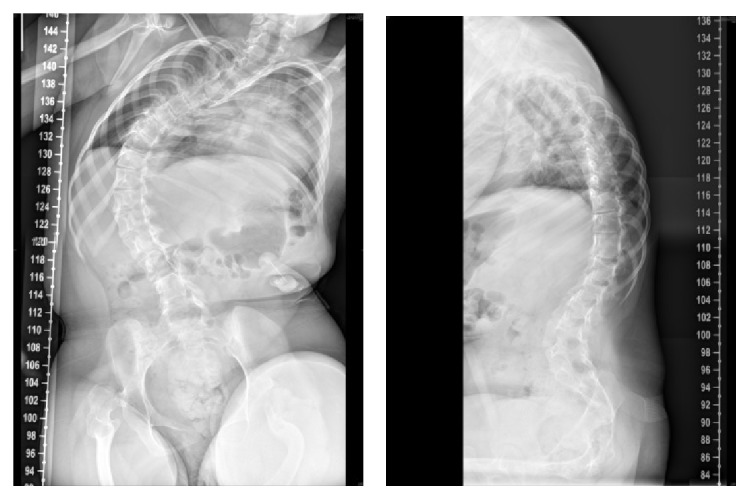
Preoperative radiographs showing a 100-degree scoliosis deformity in a severely involved cerebral palsy patient. The patient has pelvic obliquity of 15 degrees with significant coronal decompensation. Patient is nonambulatory with unilateral hip dislocation.

In our second case, a 13.5-year-old girl with a 100° right-sided main thoracic curve (T6-L1) underwent minimally invasive surgery for correction of her spinal deformity. She presented with cerebral palsy in addition to a history of seizures, developmental delay, and a recent diagnosis of obstructive sleep apnea (Figure 9).

**Figure 10:**
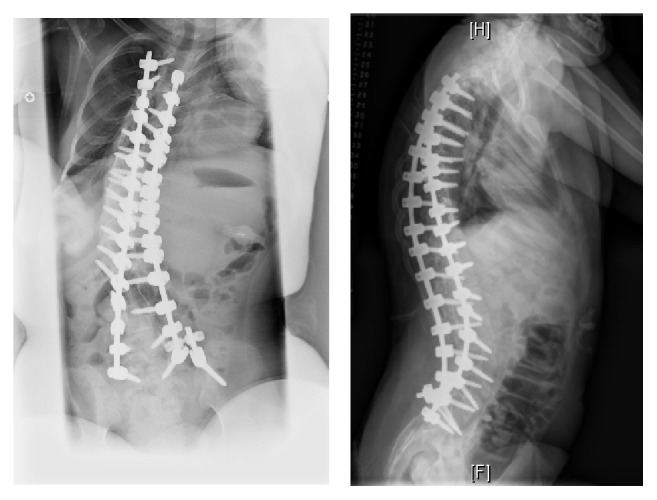
Immediate postoperative radiograph images showing spinal fusion and instrumentation from T3-S1 with significant improvement of the deformity. Unilateral pelvic fixation was carried out in this patient.

Moreover, the patient is nonverbal, nonambulatory, feeding tube dependent, and asthmatic. Using a similar approach, the patient was instrumented with pedicle screws from T3-S1. No remarkable neuromonitoring changes occurred during the surgery. Total surgical duration was 7 hours with an estimated blood loss of 800 mL. Patient received 2 units of packed red blood cells and 1 unit of platelets intraoperatively. Patient was extubated POD #1 and was transferred to the floor. Pain management was similar to the previously described case. Patient was discharged on POD #5. The postoperative X-rays demonstrate pelvic fixation and show good correction in the coronal and sagittal planes (Figure 10).

## 4. Discussion

Patients with neuromuscular scoliosis experience higher rates of complications, prolonged ICU stay, and increased blood loss. Jain et al. grouped 617 patients with different diagnosis to assess the relationship between diagnosis and blood loss in children undergoing posterior spinal fusion surgery. They found that patients with cerebral palsy and other neuromuscular disorders had a significantly higher normalized blood loss than patients with idiopathic scoliosis [7]. Edler et al. [5] compared 163 neuromuscular patients with 80 nonneuromuscular patients. They found that 65% of neuromuscular patients lost more than 50% of their estimated blood volume. Modi et al. reported a mean blood loss of 3221 ± 1711 mL in their review of 50 patients with neuromuscular scoliosis who underwent posterior spine fusion using all pedicle screw construct [8]. Twenty of their patients had blood loss of 3500 mL or more, which they found to be a clear determinant of postoperative complications[8]. Tsirikos and Mains reviewed 45 consecutive patients with quadriplegic cerebral palsy who underwent spinal fusion using pedicle screw instrumentation. Thirty-eight patients underwent posterior spine fusion, while 7 underwent staged anterior and posterior spine fusion. They reported an average correction of 74.1%, overall with average blood loss of 0.8 blood volumes, ICU stay of 3.5 days, and hospital stay of 17.6 days in posterior-only group. In anteroposterior group, the average blood loss was 0.9 blood volumes, ICU stay was 8.9 days, and hospital stay was 27.4 days [9]. Tsirikos et al. carried out a retrospective review of 287 patients treated with the unit rod instrumentation with 242 posterior-only and 45 anterior-posterior procedures. They reported an average 68% correction of the deformity. In posterior-only fusion, the average blood loss was 2.8 L, ICU stay was 4.9 days, and the hospital stay was 19.6 days. In combined procedures, the average blood loss was 3.4 L, ICU stay was 6.7 days, and the hospital stay was 24.5 days [10]. Hammett et al. retrospectively reviewed 11 patients with Rett syndrome who underwent scoliosis surgery. The average age of the cohort was 12. Eight of their patients suffered one or more significant complications with an average inpatient stay of 18.2 days [11]. Rumbak et al. reviewed patients with Rett syndrome undergoing scoliosis surgery in regard to rates of respiratory failure and rates of ventilator-acquired pneumonia in comparison to patients with neurologic scoliosis and adolescent idiopathic scoliosis. There were 133 patients with adolescent idiopathic scoliosis, 48 patients with neurologic scoliosis, and 8 patients with Rett syndrome. They found that patients with Rett syndrome undergoing scoliosis surgery have higher rates of respiratory failure and longer ventilation times in the postoperative period when compared with both adolescent idiopathic scoliosis and neurologic scoliosis patients [12].

Considering the reported advantages of the MIS technique in AIS and adult scoliosis surgery, it is intuitive to apply the benefits in neuromuscular scoliosis. Certainly less blood loss and pain can impact the amount of pain medications, fluids, and even the duration of intubation in these compromised children. However, MIS technique in neuromuscular scoliosis has thus far not been reported largely due to concern for prolonged surgery and anesthesia, which can increase the blood loss, fluids, and risk of infection. Also it is challenging to pass a curved rod over 12-13 segments through small incisions and to pass it over 16–18 segments and the pelvic fixation appears quite daunting. We have therefore modified the MIS for AIS approach and have utilized it in neuromuscular patients. Instead of multiple skin incisions, we now use a single midline incision that follows the curve. After this the dissection is between the multifidus medially and longissimus laterally. Since this plane exists naturally, the dissection involves separation of the muscles and is quite bloodless. Occasionally, a small leash of blood vessel is seen on the surface of longissimus and can be coagulated preemptively. Instead of making separate stab incisions in the fascia, which is done in the MIS approach, we make a longitudinal incision along the muscle plane. We find that multiple stab incisions made over adjacent levels tend to coalesce leading to a long incision in the fascia along the muscle plane. Thus single long fascial incision makes the dissection neat and efficient. In the thoracic spine, the crossing fibers of latissimus dorsi and trapezius overlie the thoracodorsal fascia and they need to be dissected to allow access to the facet joint. Generally the facet joints lie 1.5–2 cm from the midline on the concave side and 2–2.5 cm on the convex side. As the dissection proceeds caudally, this distance becomes smaller. In the lumbar spine the facet joints lie more superficial to the transverse process and are easily palpable, whereas in the thoracic spine the transverse process is more prominent. Thus we prefer to start in the lumbar and thoracolumbar spine and extend cephalad following the overlapping spine anatomy to expose thoracic spine facets.

It can be argued that the size of the skin incision does not determine minimally invasive technique. Minimally invasive approach in this patient population refers to preservation of tissues, utilization of the preexisting muscle planes, and less disruptive and traumatic dissection. This leads to a lesser amount of pain and blood loss, which is determinant of complications in this group of patients [8]. We have utilized the MIS technique on curves up to 100 degrees and even in children with severe disease involvement. For curves >70 degrees or smaller but less flexible curves, we have utilized previously described transforaminal type osteotomy/facet resection [6]. The entire facet, facet joint capsule, and most ligamentum flavum can be excised, which increases the flexibility and can be carried out at multiple levels. The midline ligamentous and bony structures are preserved [6].

The highlight of this surgical technique paper is the immense perioperative benefit it accrues to children with neuromuscular scoliosis who are otherwise at a much higher risk for morbidity and mortality. The weaknesses are the short-term follow-up and concerns of loss of correction even nonunion. We are presenting this as a surgical technique paper with two case reports showing significant advantages for a challenged population. Our technique has lower blood loss, lesser transfusion risk, and comparable operative time. In addition patients have lower pain medication requirements, stay in the ICU for one day, and can be discharged home in 4–6 days. These advantages are due to better soft tissue preservation, utilizing intermuscular planes for dissection which decreases bleeding and pain. This in turn helps with ICU and hospital stay and pain medications. These combined benefits are much superior to those which have been previously reported and are worthy of reporting even with short-term follow-up. This technique paper hence focuses on the surgical approach and perioperative benefits. The pseudarthrosis rate previous to pedicle screw fixation was around 10% [13]. With pedicle screw fixation nonunion is rare and is usually associated with deep infection. Our technique is no different than the standard method in terms of fixation and is comparable to fusion area, but its perioperative benefits are far superior and can potentially change the outcomes in neuromuscular scoliosis which are otherwise riddled with major medical complications.

Our results indicate that good correction in coronal and sagittal planes can be achieved safely. In curves that are flexible, 75–80% correction can be achieved. However the greatest benefit of this technique is less blood loss, shorter PICU and hospital stay, earlier mobilization, and less pain and need for pain medication [1]. The operative time needed to complete this surgery is comparable to standard scoliosis surgery [1, 2].

## 5. Conclusion

Minimally invasive approach seems to provide the greatest benefits to patients with neuromuscular scoliosis in terms of blood loss, pain, and ICU and hospital stay. A longer-term study is currently underway at our institution.
